# Prenatal ethanol exposure in mice phenocopies *Cdon* mutation by impeding Shh function in the etiology of optic nerve hypoplasia

**DOI:** 10.1242/dmm.026195

**Published:** 2017-01-01

**Authors:** Benjamin M. Kahn, Tanya S. Corman, Korah Lovelace, Mingi Hong, Robert S. Krauss, Douglas J. Epstein

**Affiliations:** 1 Department of Genetics, Perelman School of Medicine, University of Pennsylvania, Philadelphia, Pennsylvania 19104, USA; 2 Department of Developmental and Regenerative Biology, Icahn School of Medicine at Mount Sinai, New York, New York 10029, USA

**Keywords:** Optic nerve hypoplasia, Shh, Ethanol, Cdon, Septo-optic dysplasia

## Abstract

Septo-optic dysplasia (SOD) is a congenital disorder characterized by optic nerve, pituitary and midline brain malformations. The clinical presentation of SOD is highly variable with a poorly understood etiology. The majority of SOD cases are sporadic, but in rare instances inherited mutations have been identified in a small number of transcription factors, some of which regulate the expression of *Sonic hedgehog* (*Shh*) during mouse forebrain development. SOD is also associated with young maternal age, suggesting that environmental factors, including alcohol consumption at early stages of pregnancy, might increase the risk of developing this condition. Here, we address the hypothesis that SOD is a multifactorial disorder stemming from interactions between mutations in Shh pathway genes and prenatal ethanol exposure. Mouse embryos with mutations in the Shh co-receptor, *Cdon*, were treated *in utero* with ethanol or saline at embryonic day 8 (E8.0) and evaluated for optic nerve hypoplasia (ONH), a prominent feature of SOD. We show that both *Cdon^−/−^* mutation and prenatal ethanol exposure independently cause ONH through a similar pathogenic mechanism that involves selective inhibition of Shh signaling in retinal progenitor cells, resulting in their premature cell-cycle arrest, precocious differentiation and failure to properly extend axons to the optic nerve. The ONH phenotype was not exacerbated in *Cdon^−/−^* embryos treated with ethanol, suggesting that an intact Shh signaling pathway is required for ethanol to exert its teratogenic effects. These results support a model whereby mutations in *Cdon* and prenatal ethanol exposure increase SOD risk through spatiotemporal perturbations in Shh signaling activity.

## Abstract (precis)

**Summary:** *In utero* exposure to ethanol causes defects in optic nerve formation by interfering with Shh signaling activity in the growth and differentiation of retinal progenitor cells in the developing mouse eye.

## INTRODUCTION

Septo-optic dysplasia (SOD) is a clinically heterogeneous disorder that is diagnosed on the presence of at least two of the following conditions: optic nerve hypoplasia (ONH), hypopituitarism and absence of the septum pellucidum (Webb and Dattani, 2010). The severity of these features varies widely in SOD, which has an incidence of 1 in 10,000 live births (Patel et al., 2006). ONH is the most common finding in SOD, and manifests as a thinning of the optic nerve as it exits the eye, resulting in insufficient photo-transduction to the brain and, in many instances, blindness (Morishima and Aranoff, 1986; Cemeroglu et al., 2015). Variable pituitary dysfunction, including isolated growth hormone deficiency, central hypothyroidism, and panhypopituitarism is also observed in individuals with SOD, with decreased levels of one or more pituitary hormones being diagnosed by two years of age (Cemeroglu et al., 2015). Cognitive delay and seizure disorders are also frequently seen in SOD.

The cause of SOD is poorly understood. Most cases are idiopathic, but in rare instances (<1%) inherited mutations have been described in a small number of transcription factors (*SOX2*, *SOX3*, *HESX1*, *OTX2*, *TCF7L1*) expressed during embryonic brain development (McCabe et al., 2011; Gaston-Massuet et al., 2016). The high phenotypic variability, coupled with its sporadic nature, suggest that SOD might be influenced by a combination of environmental and genetic factors.

Insight into the pleiotropic nature of the SOD phenotype was recently realized from the study of a conditional mouse mutant lacking Shh in the developing hypothalamus (*Shh^Δhyp^*). *Shh^Δhyp^* mutants display optic nerve and pituitary defects with similarities to SOD in humans (Zhao et al., 2012). The eye and pituitary develop in close proximity to the source of SHH in the anterior hypothalamus and depend on this signal for formation of the optic disc, from where the optic nerve exits the eye, and for coordinating pituitary morphogenesis. These findings raise the possibility that reduced SHH expression and or signaling activity from the hypothalamus might underlie the pathogenesis of SOD in humans. In support of this hypothesis, *SOX2* and *SOX3* – two SOD-associated genes – were shown to be dose-dependent regulators of *SHH* transcription that directly bind and activate a long-range *SHH* forebrain enhancer (Zhao et al., 2012).

Nonetheless, loss-of-function mutations in *SHH* are not associated with SOD (Paulo et al., 2015; Gregory et al., 2015), but instead are known to cause another brain malformation, holoprosencephaly (HPE), with partially overlapping features to SOD (Roessler et al., 1996). HPE results from imperfect separation of the cerebral hemispheres and craniofacial structures as a result of reduction in SHH signaling from the prechordal plate, a transient embryonic tissue required for early aspects of forebrain development, including the specification of the hypothalamic territory (Chiang et al., 1996). Therefore, HPE and SOD could be distinguished by the timing and location of *SHH* signal disruption, with an early loss of SHH from the prechordal plate giving rise to HPE, and a slightly later absence of SHH from the presumptive hypothalamus resulting in SOD.

The SHH pathway has many roles during eye development. Early functions include separation of the eye fields and patterning of the optic cup (Chiang et al., 1996). At later stages, Shh secreted from retinal ganglion cells (RGCs) controls the proliferation of multipotent retinal progenitor cells (RPCs), the timing of their differentiation, as well as the guidance of RGC axons out of the eye (Wang et al., 2005; Kolpak et al., 2005; Sanchez-Camacho and Bovolenta, 2008; Stacher Hörndli and Chien, 2012). Mice lacking Shh in RGCs display ONH resulting from a failure in optic disc formation (Dakubo et al., 2003). Thus, ONH can arise by interfering with SHH signaling from two independent sources, anterior hypothalamus and RGCs, at distinct stages of eye development.

Epidemiological studies indicate that SOD associates with young maternal age and primiparity (Haddad and Eugster, 2005; Murray et al., 2005; Garcia-Filion and Borchert, 2013; Cemeroglu et al., 2015). How these risk factors contribute to the etiology of SOD is unknown, but they might be linked to adverse maternal behavior during early stages of pregnancy (Garcia-Filion and Borchert, 2013). For instance, several clinical features of fetal alcohol syndrome overlap with HPE and SOD, suggesting that prenatal ethanol exposure might increase the risk of both conditions, depending on the timing of the insult (Sulik et al., 1981; Strömland, 1987; Coulter et al., 1993; Ashwell and Zhang, 1994; Blader and Strähle, 1998; Hellström, 1999; Ribeiro et al., 2007; Aoto et al., 2008; Loucks and Ahlgren, 2009; Lipinski et al., 2010, 2012; Zhang et al., 2011).

The SHH signaling pathway is a key target of prenatal ethanol exposure and its perturbation explains much of the HPE-like phenotype observed in animal models of this condition (Ahlgren et al., 2002; Li et al., 2007; Higashiyama et al., 2007; Aoto et al., 2008). Interestingly, mouse embryos with mutations in Shh pathway genes that have no, or minimal, phenotypic consequence on their own, show a profound increase in the penetrance and severity of HPE when exposed to sub-teratogenic doses of ethanol (Hong and Krauss, 2012; Kietzman et al., 2014). The synergy between these genetic and environmental risk factors for HPE is dependent on the timing of ethanol administration during pregnancy, with a strong interaction observed at embryonic day 7 (E7.0), coinciding with a disruption in Shh signaling from the prechordal plate (Hong and Krauss, 2012).

On the basis of these studies, we postulate that SOD is a multifactorial condition that results from interactions between genetic and environmental risk factors acting at slightly later stages of forebrain development than those that cause HPE. To test this hypothesis and better define the relationship between ethanol intake, Shh signaling and SOD, we examined eye development in mouse embryos with mutations in the Shh co-receptor, *Cdon*, that were exposed *in utero* to either ethanol or saline at E8.0. Wild­-type embryos treated with ethanol phenocopied *Cdon^−/−^* mutants treated with saline in the manifestation of ONH by selectively impeding Shh signaling activity in RPCs. The combination of *Cdon* mutation and ethanol exposure did not worsen the ONH phenotype, indicating that this gene–environment interaction is not additive or synergistic. These results support a model whereby mutations in *Cdon* and prenatal ethanol exposure are risk factors for SOD and HPE through temporally and spatially distinct perturbations in Shh signaling activity.

## RESULTS

We followed a previously validated protocol for prenatal ethanol exposure (see Materials and Methods) to determine whether *Cdon^−/−^* embryos were sensitive to ethanol-induced ONH, a prominent feature of SOD. All mice described in this study were maintained on a 129S6/SvEvTac genetic background, which is largely impervious to the HPE-associated phenotypes caused by *Cdon* mutation or ethanol exposure observed in other mouse strains (Zhang et al., 2006; Downing et al., 2009; Hong and Krauss, 2012). Pregnant *Cdon^+/−^* females that were time-bred with *Cdon^+/−^* males received intraperitoneal injections of ethanol (3.48 g/kg) or saline at E8.0 and again four hours later. This embryonic stage was chosen because it was subsequent to the HPE-critical period at E7.0, allowing us to address the temporal specificity of gene–environment interactions in the etiology of SOD.

### *Cdon* mutation and prenatal ethanol exposure independently cause ONH

*Cdon* is expressed at early stages of eye development (E9-E11.5), including progenitors of the neural retina and lens vesicle (Zhang et al., 2009). *Cdon^−/−^* and wild-type embryos were harvested at E14.5, cryo-sectioned along the coronal plane of their heads, and immunostained for neurofilament. No gross abnormalities in the size or structure of the brain were observed between wild-type and *Cdon^−/−^* embryos in either the ethanol or saline treatment groups. Moreover, none of the prominent eye defects displayed by *Cdon^−/−^* mutant embryos on the C57BL/6 genetic background, including coloboma, microphthalmia and lens dysmorphology (Zhang et al., 2009) were detected in any of the 129S6 embryos (129S6.*Cdon^−/−^*), consistent with the strain specificity of these phenotypes.

**Fig. 1.**
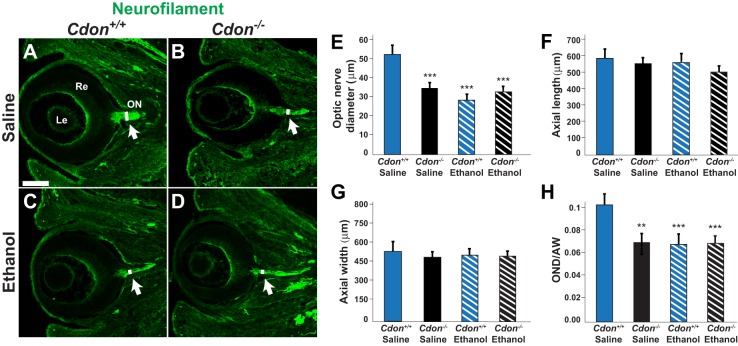
***Cdon* mutation and ethanol exposure independently cause optic nerve hypoplasia.** (A-D) Immunostaining for neurofilament (green) on transverse sections through the eye at E14.5 labels the optic nerve (arrow). Compared with (A) saline­-treated wild-type (*Cdon^+/+^*) embryos (*n*=9), the diameter of the optic nerve (white line) is significantly reduced in (B) saline­-treated *Cdon^−/−^* mutants (*n*=8), (C) ethanol-treated wild-type embryos (*n*=9), and (D) ethanol-treated *Cdon^−/−^* mutants (*n*=9). Scale bar: 200 µm. (E-H) Quantification of (E) optic nerve diameter, (F) axial length of eye, (G) axial width of eye, and (H) optic nerve diameter (OND) normalized to axial width (AW) of the eye. Error bars represent s.d. **P<0.01, ***P<0.001 by Student's *t*-test.

To assess the embryos for ONH, the diameter of the optic nerve was measured at the level of the optic disc. *Cdon^−/−^* embryos treated with saline showed a 39% reduction in optic nerve diameter (mean±s.d., 32.26±1.83 μm, *n*=9, *P*<0.001) compared with control littermates (52±3.47 μm, *n*=8) (Fig. 1A,B,E). This difference was significant after normalizing for eye size (Fig. 1F-H). Wild­-type embryos exposed to ethanol showed a similar reduction in optic nerve diameter (29.7±2.14 μm, *n*=9, *P*<0.001) compared with saline-treated controls (Fig. 1A,C). This result was unexpected given that 129S6 embryos were thought to be resistant to ethanol-mediated teratogenicity (Downing et al., 2009; Hong and Krauss, 2012), although the optic nerve was not examined in these prior studies. The combination of *Cdon* mutation and prenatal ethanol exposure did not exacerbate the ONH phenotype compared with embryos with either condition alone. Ethanol-treated *Cdon^−/−^* embryos showed a 37% decrease in optic nerve width (33±2.4 μm, *n*=8, *P*<0.001) compared with saline­-treated controls (Fig. 1A,D), which was a similar reduction to that seen in saline-treated *Cdon^−/−^* embryos and ethanol-treated wild-type embryos (Fig. 1A-E). These data indicate that *Cdon* mutation and prenatal ethanol exposure both contribute to the etiology of ONH and that additional risk factors, such as genetic background (129S6 versus C57BL/6) and timing of ethanol exposure (E7.0 versus E8.0), influence the phenotypic outcome of ONH versus HPE.

### Formation of the optic disc is not disturbed in *Cdon^−/−^* and ethanol-exposed embryos

**Fig. 2.**
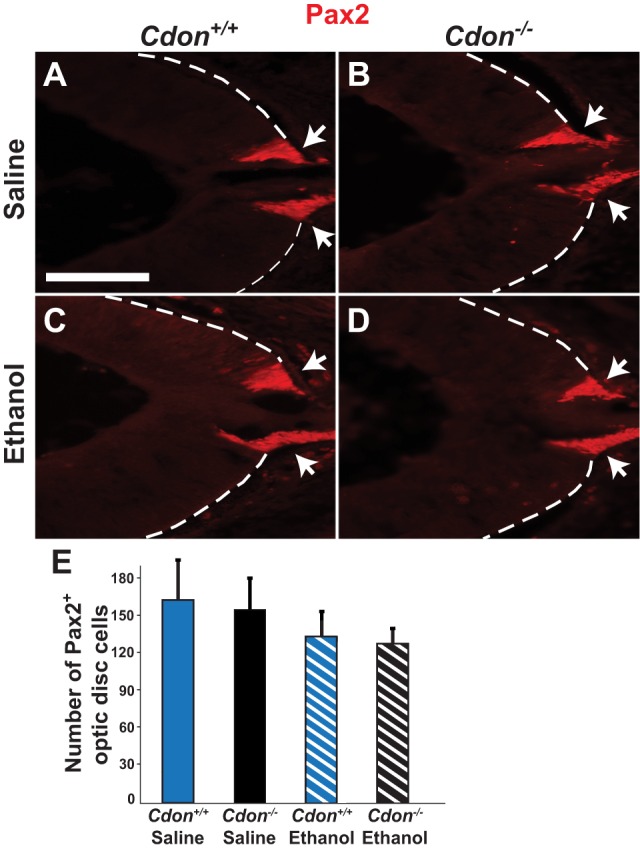
**The optic disc is not compromised in *Cdon^−/−^* or ethanol-treated embryos.** (A-D) Immunostaining for Pax2 on transverse sections through the eye at E14.5 marks the optic disc (arrows). No significant differences were observed in the average number of Pax2^+^ optic disc cells per section from (A) saline­-treated wild-type (*Cdon^+/+^*) embryos (*n*=6), (B) saline­-treated *Cdon^−/−^* mutants (*n*=8), (C) ethanol-treated wild-type embryos (*n*=8), and (D) ethanol-treated *Cdon^−/−^* mutants (*n*=8). Scale bar: 200 µm. (E) Quantification of Pax2^+^ optic disc cells. Error bars represent s.d. Student's *t*-test.

The optic nerve exits the eye through the optic disc, which forms at the juncture of the optic stalk and cup. ONH can arise from defects in optic disc formation or from a deficit in the number of RGC axons that make up the optic nerve (Deiner et al., 1997; Dakubo et al., 2003; Zhao et al., 2012). To distinguish between these two possibilities, we evaluated the expression of Pax2 in the optic disc of *Cdon^−/−^* and wild-type embryos that were exposed *in utero* to either saline or ethanol at E8.0 of gestation. No significant differences were observed between the number of Pax2^+^ cells in embryos from the experimental and control groups (Fig. 2), thus excluding major defects in optic disc formation as a likely explanation for the ONH phenotype in either of these mouse models.

### Shh-dependent proliferation of RPCs is compromised in *Cdon*^−/−^ and ethanol-treated embryos

The absence of a synergistic interaction between *Cdon^−/−^* mutation and prenatal ethanol exposure in the manifestation of ONH suggested that both insults might be disrupting a common or parallel signaling pathway(s) important for eye development. Shh is the most likely pathway to be compromised in these mouse models of ONH given the established role of Cdon as a Shh co-receptor, the essential function of Shh in RPC proliferation, and the negative influence of ethanol on Shh pathway activation in a variety of developing tissues (Ahlgren et al., 2002; Wang et al., 2005; Zhang et al., 2006; Tenzen et al., 2006; Li et al., 2007; Aoto et al., 2008; McLellan et al., 2008; Allen et al., 2011; Hong and Krauss, 2013).

**Fig. 3.**
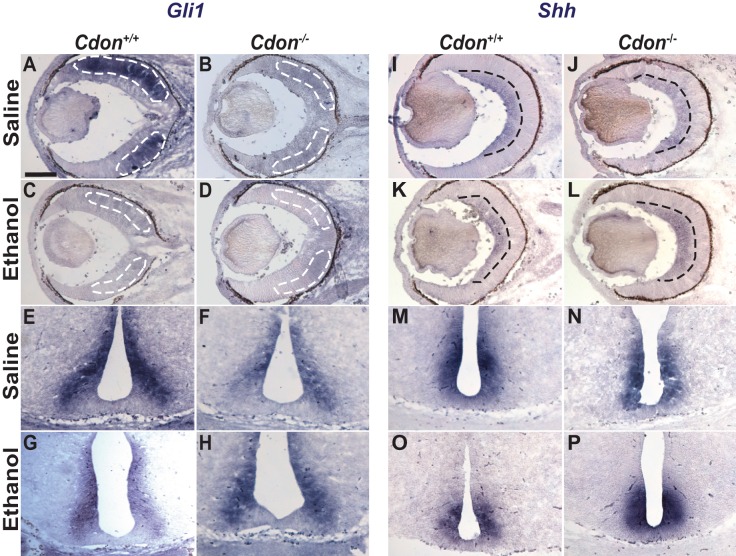
**Selective reduction of *Gli1* expression in the eyes of *Cdon^−/−^* and ethanol-treated embryos.** *In situ* hybridization for *Gli1* (A-H), and *Shh* (I-P) on transverse sections through the eye (A-D,I-L) and hypothalamus (E-H,M-P) of E14.5 wild-type (*Cdon^+/+^*) and *Cdon^−/−^* embryos treated with saline or ethanol at E8.0. *Gli1* expression is detected in retinal progenitor cells (RPCs, area marked by dotted white line) of (A) saline­-treated wild-type embryos (*n*=5). *Gli1* expression is markedly reduced in RPCs of (B) saline­-treated *Cdon^−/−^* mutants (*n*=5), (C) ethanol-treated wild-type embryos (*n*=9), and (D) ethanol-treated *Cdon^−/−^* mutants (*n*=5). No differences were observed in the expression of *Gli1* in the hypothalamus between genotypes or treatment groups (E-H). No differences were observed in the level of *Shh* expression in retinal ganglion cells (RGCs, area marked by dotted black line, I-L) or the hypothalamus (M-P) between genotypes or treatment groups. Scale bar: 200 µm.

To evaluate the integrity of Shh signaling we assessed *Gli1* expression, a reliable readout of Shh pathway activation (Marigo et al., 1996), on sections through the eye at E14.5. Wild-type embryos treated with saline showed robust expression of *Gli1* in the RPC layer of the developing eye at E14.5 (Fig. 3A). In comparison, *Gli1* was markedly reduced in the RPCs of wild-type and *Cdon^−/−^* embryos exposed to ethanol at E8.0, as well as *Cdon^−/−^* embryos treated with saline (Fig. 3A-D). The downregulation of *Gli1* seemed to be specific to the eye as an adjacent domain of expression in the anterior hypothalamus was unaffected across genotypes and treatment groups (Fig. 3E-H). Moreover, *Shh* expression was not compromised in the eye or hypothalamus of any of the embryos (Fig. 3I-P), suggesting that both *Cdon* mutation and prenatal ethanol exposure were acting directly on some aspect of RPC development downstream of Shh.

**Fig. 4.**
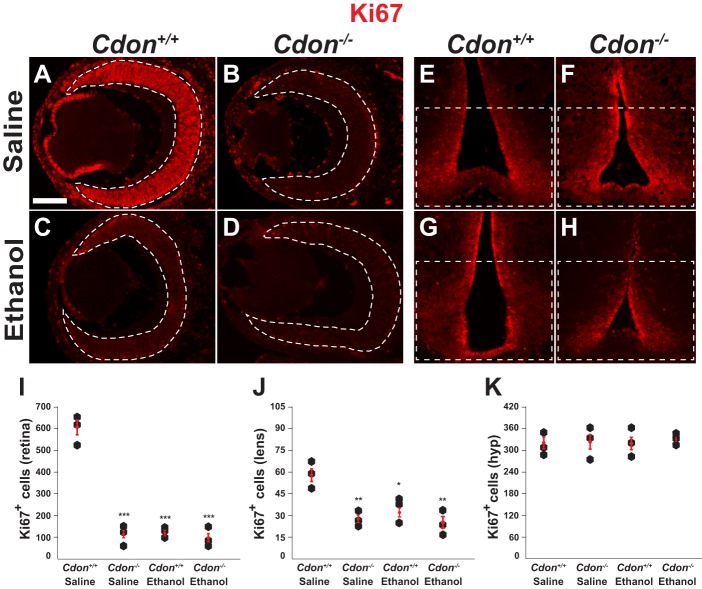
**Reduced proliferation of RPCs in *Cdon^−/−^* and ethanol-treated embryos.** (A-H) Immunostaining for Ki67 on transverse sections through the eye (A-D) and hypothalamus (E-H) of E14.5 embryos labels proliferating progenitors. (A) The majority of retinal progenitor cells (RPCs, area marked by dotted white line) in saline­-treated wild-type (*Cdon^+/+^*) embryos (*n*=3), are marked by Ki67. The number of Ki67^+^ RPCs is significantly reduced in (B) saline­-treated *Cdon^−/−^* embryos (*n*=3), (C) ethanol-treated wild-type embryos (*n*=3), and (D) ethanol-treated *Cdon^−/−^* embryos (*n*=3). No differences in the number of Ki67^+^ cells in the ventricular layer of the ventral hypothalamus (boxed area) were observed between genotypes or treatment groups (E-H). Scale bar: 200 µm. (I-K) Quantification of Ki67^+^ cells in (I) retina, (J) lens and (K) hypothalamus sections across all experimental groups. Error bars represent s.d. *P<0.05, **P<0.01, ***P<0.001 by Student's *t*-test.

Shh signaling maintains RPCs in a mitotically active state until they are poised to differentiate into RGCs (Zhang and Yang, 2001; Wang et al., 2005). Therefore, we next determined whether the downregulation in Shh signaling observed in *Cdon^−/−^* and ethanol-treated embryos compromised the growth and differentiation properties of RPCs. The proliferation marker Ki67 labeled 670 RPCs per section (*n*=3) in saline­-treated wild-type embryos at E14.5 (Fig. 4A,I). By contrast, a drastic reduction in the number of Ki67-positive RPCs was observed in *Cdon^−/−^* embryos treated with ethanol (88 RPCs/section, *n*=3, *P*<0.001) or saline (117 RPCs/section, *n*=3, *P*<0.001), as well as wild-type embryos exposed to ethanol (113 RPCs/section, *n*=3, *P*<0.001) (Fig. 4A-D,I). Despite the significant reduction in Ki67 staining, trace amounts were still detected in *Cdon^−/−^* and ethanol-treated embryos upon increased exposure times. Reduced proliferation was also noted in the lens epithelium of *Cdon^−/−^* and ethanol-treated embryos (Fig. 4J), as described previously (Zhang et al., 2009). The proliferation defects seem to be specific to the eye as no significant differences were detected in the number of Ki67-positive neural progenitors in adjacent brain regions from either genotype or treatment group (Fig. 4E-H,K). These results suggest that the failure of RPCs to respond to Shh signaling in both *Cdon^−/−^* and ethanol-treated embryos at E8.0 compromises their ability to replicate, in agreement with other studies of Shh signaling in the eye (Wang et al., 2005).

### Precocious differentiation of RGCs in *Cdon*^−/−^ and ethanol-treated embryos

**Fig. 5.**
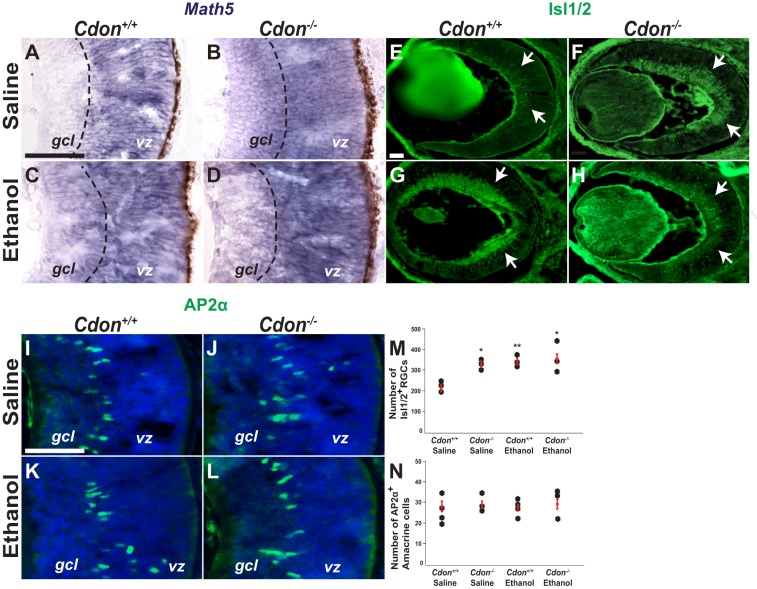
**Precocious differentiation of RGCs in *Cdon^−/−^* and ethanol-treated embryos.** (A-D) *In situ* hybridization for *Math5* on transverse sections through the eye at E14.5 labels postmitotic progenitors in the ventricular zone (vz) of control embryos (*n*=3) (A). *Math5* expression expands into the ganglion cell layer (gcl) of *Cdon^−/−^* and ethanol-treated embryos (*n*=3 for each experimental group) (B-D). Dashed line marks the vz–gcl boundary. (E-H) Immunostaining for Isl1/2 on transverse sections through the eye of E14.5 embryos primarily labels differentiating retinal ganglion cells (RGCs, arrows). Compared with (E) saline­-treated wild-type (*Cdon^+/+^*) embryos (*n*=3), the number of Isl1/2^+^ RGCs is significantly increased in (F) saline­-treated *Cdon^−/−^* mutants (*n*=3), (G) ethanol-treated wild-type embryos (*n*=3), and (H) ethanol-treated *Cdon^−/−^* mutants (*n*=3). (I-L) Immunostaining for AP-2α in amacrine cells. No significant differences were observed between the average number of AP-2α^+^ amacrine cells per section from (I) saline­-treated wild-type (*Cdon^+/+^*) embryos (*n*=4), (J) saline­-treated *Cdon^−/−^* mutants (*n*=3), (K) ethanol-treated wild-type embryos (*n*=4), and (L) ethanol-treated *Cdon^−/−^* mutants (*n*=3). Scale bar: 50 µm. (M,N) Quantification of cells expressing Isl1/2 (M) and AP-2α (N). Error bars represent s.d. *P<0.05, **P<0.01 by Student's *t*-test.

To determine if the differentiation of RPCs was affected by their premature cell-cycle exit we assessed expression of *Math5* (also known as *Atoh7*), a bHLH transcription factor required at the onset of RGC differentiation (Wang et al., 2001). In control embryos, *Math5* expression was confined to postmitotic progenitors in the ventricular zone. By contrast, in *Cdon^−/−^* and ethanol-treated embryos, *Math5* expression extended from the ventricular zone into the ganglion cell layer (Fig. 5A-D). This observation is similar to the previous report of expanded *Math5* expression in mouse mutants that lack Shh signaling in the eye (Sakagami et al., 2009), and suggests that loss of Shh*-*dependent RPC proliferation might be associated with precocious differentiation of RPCs.

RGCs are the earliest born retinal cell type originating from a subset of RPCs expressing Math5 (Feng et al., 2010; Brzezinski et al., 2012). The LIM homeobox transcription factor Isl1 functions downstream of Math5, and in conjunction with the POU domain protein Pou4F2 promotes RGC differentiation (Mu et al., 2008; Pan et al., 2008; Prasov and Glaser, 2012; Wu et al., 2015). We evaluated the status of RGC differentiation in *Cdon^−/−^* and ethanol-treated embryos by immunostaining for Isl1. At E14.5, RGCs are still early in their differentiation as evidenced by the sparse labeling of Isl1 in saline­-treated wild-type embryos (225 cells/section, *n*=3) (Fig. 5E). However, the number of Isl1-positive cells was increased by 32% in wild-type (329 cells/section, *n*=3, *P*<0.05) and *Cdon^−/−^* (338 cells/section, *n*=3, *P*<0.01) embryos exposed to ethanol at E8.0, as well as in saline­-treated *Cdon^−/−^* mutants (349 cells/section, *n*=3, *P*<0.05) (Fig. 5E-H,M). Although Isl1 is not exclusively expressed by RGCs, we did not observe significant differences in the number of other early-born retinal progenitors, such as amacrine cells expressing AP-2α (also known as Tfap2a), between controls and treatment groups, suggesting that the precocious differentiation was limited to RGCs (Fig. 5I-L,N).

These data suggest that the loss of Shh signaling in *Cdon^−/−^* and ethanol-treated embryos results in the precocious differentiation of RGCs, which would likely deplete the pool of non-proliferating RPCs over time (Wang et al., 2005). The significant thinning of the optic nerve in experimental embryos likely results from the failure of these prematurely differentiating RGCs to properly extend axons to the optic disc, a premise that is supported by a previously characterized role for Shh in regulating the guidance of RGC axons (Sanchez-Camacho and Bovolenta, 2008). Taken together, our results demonstrate that prenatal ethanol exposure at E8.0 phenocopies 129S6.*Cdon^−/−^* mutant embryos in the manifestation of ONH by selective interference with Shh-dependent expansion and differentiation of RPCs in the eye.

## DISCUSSION

### Ethanol and *Cdon* mutation impede Shh signaling in RPCs to cause ONH

**Fig. 6.**
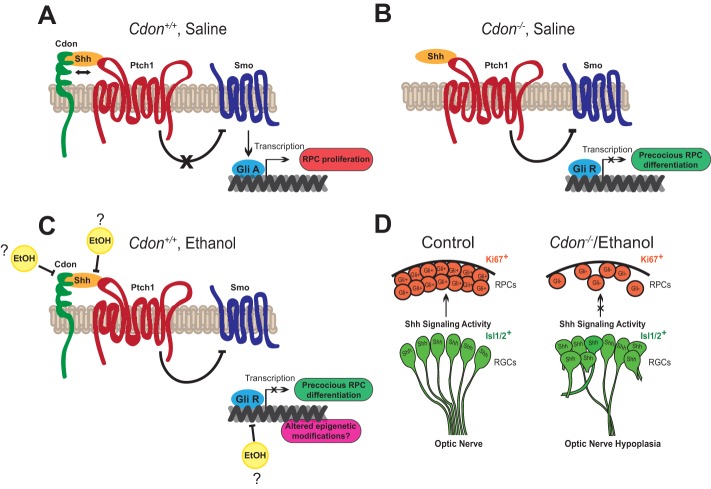
**Model depicting the influence of *Cdon* mutation and ethanol exposure on Shh signaling activity in the developing eye.** (A) In wild-type embryos (E14.5), the binding of Shh to Cdon and Ptch1 releases the inhibition on smoothened (Smo), facilitating the transcription by Gli activator (GliA) of target genes involved in RPC proliferation. (B) In the eyes of 129S6.*Cdon^−/−^* embryos, there is persistent inhibition of Smo by Ptch1, even in the presence of Shh, causing Gli repressor (GliR) to block transcription of genes involved in RPC proliferation, resulting in precocious RPC differentiation. (C) Ethanol exposure at E8.0 interferes with Shh signaling in the eye through a variety of proposed mechanisms. The lengthy delay between ethanol exposure (E8.0) and its negative effects on Shh signaling activity (E14.5), suggests that the epigenetic landscape of Shh target genes might be modified to suppress RPC proliferation. (D) *Cdon* mutation or ethanol exposure at E8.0 impedes Shh signaling activity in RPCs resulting in optic nerve hypoplasia.

The association of SOD with young maternal age led to the hypothesis that adverse behavior, including prenatal alcohol exposure, is a predisposing factor in its etiology (Haddad and Eugster, 2005; Murray et al., 2005; Garcia-Filion and Borchert, 2013; Cemeroglu et al., 2015). Fetal exposure to alcohol causes a spectrum of developmental disorders; however, direct evidence linking ethanol to SOD has been lacking. Here, we used a mouse model to demonstrate that *in utero* exposure to ethanol at E8.0 causes ONH, the most prevalent SOD-associated phenotype. We show that ethanol causes ONH through a similar mechanism to that observed in *Cdon^−/−^* embryos, involving the inhibition of Shh signaling activity in retinal progenitor cells, which leads to their premature cell-cycle arrest, precocious differentiation, and failure to properly extend axons to the optic nerve (Fig. 6).

These data are consistent with previous studies showing that Shh secreted from RGCs is required to maintain RPCs in a proliferative state, thus preventing their differentiation (Zhang and Yang, 2001; Wang et al., 2005; Sakagami et al., 2009). RGCs also remain dependent on Shh during their maturation, as evidenced by the axonal outgrowth defects that occur upon further inhibition of Shh (Kolpak et al., 2005; Sanchez-Camacho and Bovolenta, 2008). Taken together, our findings implicate the disruption of RGC-derived Shh signaling as the pathogenic mechanism by which *Cdon* mutation and prenatal ethanol exposure cause ONH (Fig. 6D).

Interestingly, Cdon has also been reported to antagonize hedgehog (Hh) signaling in the optic vesicle of zebrafish and chick embryos (Cardozo et al., 2014). However, we did not observe any of the gain of Hh function phenotypes described in Cdon morphants, including expansion of Pax2-expressing cells in the ventral retina, or increased Hh signaling in the hypothalamic territory adjacent to the eye. Moreover, the HPE phenotype displayed by *Cdon^−/−^* mouse embryos exposed to ethanol at E7.0 was rescued by increasing Shh signaling activity (Hong and Krauss, 2013), in contrast to the decrease in Hh that restored eye patterning in Cdon morphants (Cardozo et al., 2014). The differences between our findings and those of Cardozo et al. (2014) might be related to the species in which the experiments were performed, or possibly the nature of the genetic manipulations – germline mutation versus morpholino knockdown – that in some cases might result in phenotypic differences resulting from distinct modes of genetic compensation (Rossi et al., 2015).

### Strain-dependent modifiers and timing of prenatal ethanol exposure influence Shh-related phenotypes

A particularly striking feature of our mouse model is the influence that genetic background and timing of prenatal ethanol exposure have on the variable phenotypic severity, in keeping with other studies of ethanol-induced teratogenesis (Downing et al., 2009; Lipinski et al., 2012). When bred on the 129S6/SvEvTac strain, both *Cdon^−/−^* mutants and wild-type embryos exposed to ethanol at E8.0 presented with ONH. By contrast, when raised on a C57BL/6 (C57BL/6NTac or C57BL/6J) genetic background, both *Cdon^−/−^* embryos, and wild-type embryos exposed to ethanol one day earlier at E7.0, exhibited HPE (Zhang et al., 2006; Higashiyama et al., 2007; Aoto et al., 2008; Godin et al., 2010). Thus, strain-dependent modifiers of the *Cdon*^−/−^ mutation and timing of prenatal ethanol exposure affect the spatiotemporal dynamics of Shh pathway disruption in the eye and prechordal plate, which influences the likelihood of developing ONH versus HPE, respectively.

It is intriguing that we did not detect any interaction between *Cdon*^−/−^ mutation and ethanol in the manifestation of ONH, or other SOD-related phenotypes, whereas synergy between the two insults was observed for HPE (Hong and Krauss, 2012). This finding suggests that the eye is especially vulnerable to genetic and environmental perturbations in Shh signaling, at least on the more resistant 129S6 background. Pituitary hypoplasia is another prominent feature of SOD that arises from Shh pathway disruption (Treier et al., 2001; Wang et al., 2010; Zhao et al., 2012). However, *Shh* expression in the anterior hypothalamus, which is required for pituitary morphogenesis, was not affected in the embryos analyzed in our study. Hence, more impactful perturbations in Shh signaling might be needed to compromise pituitary development, as described in other mouse models of SOD (Zhao et al., 2012; Gaston-Massuet et al., 2016).

### Effects of ethanol on Shh signaling

Another interpretation for the inability of ethanol to worsen the ONH phenotype in *Cdon^−/−^* mutants is that an intact Shh signaling pathway is required for ethanol to exert its teratogenic effect. Ethanol treatment reduces Shh signaling through diverse mechanisms, including the activation of Shh pathway antagonists (PKA), repression of Shh pathway modulators (cholesterol), and indirect consequences that decrease the survival of Shh-expressing and/or responsive cells, possibly owing to increased oxidative stress (Ahlgren et al., 2002; Li et al., 2007; Aoto et al., 2008; Zhang et al., 2011). In each of these examples the acute effect of ethanol on Shh signaling is short-lived, occurring close to the developmental stage when Shh function is required. However, in our study *Shh* is not expressed in the eye until several days after ethanol administration, suggesting that ethanol-induced alterations persist beyond the time of exposure.

One potential mechanism by which ethanol might invoke long-lasting changes in gene expression is through epigenetic modifications of DNA and chromatin structure (Kleiber et al., 2014). Acetyl-CoA is an end product of ethanol metabolism and, among its many cellular functions, serves as a substrate for histone acetylation. Stable alterations in the acetylation and methylation of histone tails at several loci were detected in the cerebral cortex of E17 mouse embryos after *in utero* ethanol exposure at E7.0 (Veazey et al., 2015). Whether these ethanol-induced changes in histone modifications alter gene expression programs that are responsible for specific developmental defects requires further experimentation. Nonetheless, these observations suggest an intriguing model in which prenatal ethanol exposure at E8.0 perturbs the epigenetic landscape leading to alterations in Shh-dependent gene expression in the eye at E14.5 (Fig. 6C).

### SOD is a multifactorial disorder

The idiopathic nature of most SOD cases suggests a multifactorial etiology to this debilitating condition, including sporadic mutations and environmental teratogens that impinge on Shh-dependent mechanisms of eye and pituitary development. Exome and whole-genome sequencing of SOD cases should assist in the identification of as-yet undiscovered genetic variants that increase disease risk. Although our study demonstrated the adverse effects of prenatal ethanol exposure on Shh signaling during eye development, other drugs, including cannabinoids and their more potent synthetic derivatives, might also contribute to disease pathogenesis by interfering with Shh signal transduction at key stages of embryonic development (Khaliullina et al., 2015; Gilbert et al., 2015). The use of drugs and alcohol at early stages of pregnancy is particularly harmful to the embryo because it coincides with a sensitive period of brain development during the first month when young mothers are often unaware of their pregnancy. A better understanding of the gene–environment interactions underlying SOD risk might improve treatment options, time to diagnosis, and public awareness of the importance for early prenatal care, even when pregnancy is inadvertent.

## MATERIALS AND METHODS

### Mice

All animal work was approved by the Institutional Animal Care and Use Committee (IACUC) at the Icahn School of Medicine at Mount Sinai and the Perelman School of Medicine, University of Pennsylvania. The animal facilities at both institutions are accredited by the Association for Assessment and Accreditation of Laboratory Animal Care International (AAALAC). Detailed methods for all mouse breeding experiments, *in utero* ethanol administration, measurements of maternal blood alcohol concentration and embryo harvest are described in Hong and Krauss (2012). Briefly, *Cdon^+/−^* mice on a 129S6/SvEvTac (129S6) background were mated for one hour in the dark and checked for the presence of a vaginal plug. The time of plug detection was designated as embryonic day 0 (E0). Pregnant female mice were injected intraperitoneally with 15 μl/g body weight of a solution of 30% ethanol in saline (3.48 g/kg) at E8.0, and again 4 h later. Saline injections were used as a control. Generation of mice with a targeted *Cdon* null allele was described previously (Cole and Krauss, 2003).

### Immunohistochemistry and *in situ* hybridization

Embryos were harvested at E14.5, fixed overnight in 4% paraformaldehyde at 4°C, washed in PBS, dehydrated through a graded ethanol series, and stored in 100% ethanol at −20°C. Embryos were rehydrated in PBS, cryoprotected in 30% sucrose overnight at 4°C, embedded in Tissue-Tek OCT Compound (Sakura Finetek USA, Inc., Torrance, CA), quick-frozen on dry ice, and cryosectioned at 16 µm. Primary antibodies used for immunohistochemistry and their dilutions are as follows: mouse anti-neurofilament (1:250, 2H3), mouse anti-islet1/2 (1:100, 39.4D5), mouse anti AP-2alpha (1:100, 5E4) were obtained from Developmental Studies Hybridoma Bank (University of Iowa, Iowa City, IA); rabbit anti-Pax2 (1:250, 71-6000, Invitrogen); mouse anti-Ki67 (1:1000, ACK02, Leica Biosystems). Detection of primary antibodies was achieved using secondary antibodies conjugated to Cy3 (1:100, 115-106-006, Jackson ImmunoResearch Laboratories) or Alexa 488 (1:100, A32723, Molecular Probes). Section *in situ* hybridization was performed with digoxygenin-UTP-labeled riboprobes essentially as described (Nissim et al., 2007). At least three to five embryos in the experimental and control groups were evaluated for each antibody or *in situ* probe.

### Quantification and statistical analysis

All cell counts were performed using the cell counter function in ImageJ (NIH) on tissue sections from at least three embryos of each experimental and control group. The width of the optic nerve was determined at its mid-point using image software in the Leica Application Suite (Leica Microsystems). The axial width and length of each eye was also determined. Eye measurements were taken from at least eight embryos of each experimental and control group that were blind to the observer. Statistical analysis was performed in GraphPad Prism using the Student's *t*-test.
